# CC360 Pearl: Interacting with Industry for the New Gastroenterologist

**DOI:** 10.1093/crocol/otaf049

**Published:** 2025-07-25

**Authors:** Benjamin Click, David Fudman

**Affiliations:** Division of Gastroenterology and Hepatology, University of Colorado School of Medicine, Aurora, CO 80209, United States; Division of Digestive and Liver Disease, University of Texas Southwestern Medical Center, Dallas, TX 75390, United States

## Pharmaceutical Company Structure 

Every company has a unique organizational structure that evolves over time. From a healthcare provider perspective, generally this structure can be simplified to 2 domains, which are segregated by compliance requirements: the areas of the company focused on product and disease state education termed the Medical domain; and the portions of the company that are primarily responsible for the commercialization, marketing and promotion of the company’s products, called the Commercial domain.

**Figure 1.**
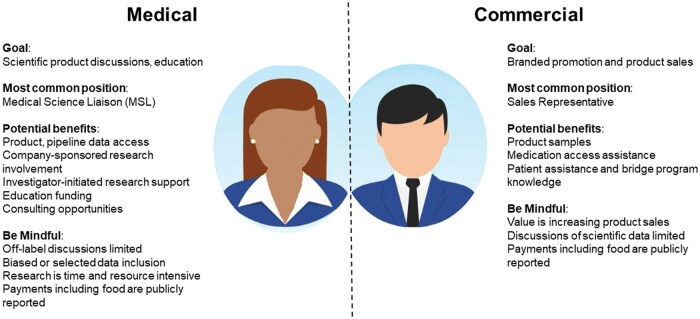
Schematic of medical and commercial domains of a typical pharmaceutical company.

Within the Medical domain, staff typically have scientific or medical background or training. The most common team member interacting with physicians are medical science liaisons (MSLs). Medical science liaisons’ interactions include educational and scientific discussions about marketed or pipeline products. The information they share is regulated by strict compliance guidelines; for example, off-label use can only be discussed at a healthcare provider’s specific request. Other roles under the medical domain include those involved in the conduct of research using the company’s product (Figure 1).

The most common Commercial staff who interact with health providers are sales representatives, who focus on the branded promotion of the company’s product. Discussions generally revolve around the merits of the product and soliciting provider feedback on access, use, and outcomes.

Some pharmaceutical companies have divisions and roles that are non-promotional with a focus on furthering disease state research, education and care, such as population health experts.

## Benefits of Industry Interaction

There are numerous potential advantages of establishing relationships with industry, but these differ by domain and representative.

### Data access

Medical science liaisons are generally well-educated on their product’s scientific data and can leverage this for provider education or citations to aid in payor appeals.

### Product access

Sales representatives supply product samples, which can be helpful when bridging a patient through an insurance coverage issue or providing rapid access to initiate therapy (individual institutional restrictions may apply). Medication access specialists can help ensure that practices have the necessary logistics in place to access their products, which is particularly pertinent for infusions. Many manufacturers provide patient assistance programs that can facilitate navigation and reduce patients’ out-of-pocket costs, and in some cases will provide no-cost drugs directly to patients when payers deny coverage (colloquially termed “bridge” programs). Notably, such assistance programs are restricted to patients with non-governmental insurance. Manufacturers can also provide self-administration training services both directly to patients as well as to providers and staff.

### Opportunities and funding for research and education

Most industry entities conduct and support research, including the pivotal registration trials programs. Participation as a site in such trials, which are often executed via a third-party research organization, has the potential to benefit both patients via access to novel therapies and practices through financial compensation. However, these trials require a multidisciplinary research team and involve substantial overhead, necessitating infrastructure, careful protocol review, and budget planning.

Industry-supported research also includes investigator-initiated research, examples of which range from randomized clinical trials, to pilot translational studies, to analyzing large third-party databases. Independent investigators propose these studies through specific company-defined pathways. Companies often publish their priorities for potential research funding; in general, their priorities align with their commercial interests. Medical science liaisons can be a key link to proposing and submitting investigator-initiated research studies or application to serve as a site for industry-sponsored studies.

Industry budgets often have funding dedicated to supporting education for healthcare providers. These can include support for advanced fellowships, Continuing Medical Education events, or visiting professorships. Funding typically requires a submission through a grant portal for each company.

### Consulting opportunities

Industry requires real-world experience and feedback to optimize product development and use. While this often occurs in informal meetings with local MSLs or sales representatives, they also take place at dedicated, topic-focused advisory boards. Participants often include “key opinion leaders,” who are often academic content and research experts; however, industry values community providers’ input as most patients receive healthcare outside an academic setting. As providers develop into content experts, invitations for additional engagements such as speakers’ bureaus may also arise. MSLs can be helpful to link interested providers to such opportunities.

## Potential Pitfalls

Although there are clear benefits that arise from interactions with pharma, these should be viewed through a lens of their potential pitfalls.

### Biased interactions

Fundamentally, all industry representatives provide value to their organization, directly or indirectly promoting the success of their products. Therefore, unbiased interactions cannot be assumed. Moreover, there are well-founded concerns regarding conscious and unconscious biases among physicians resulting from industry relationships that may influence clinical practice patterns.^1–3^

### Conflicts of interest

Because of bias concerns, there are federal and, in many cases, institutional rules that govern interactions between providers and industry. The Physician Payments Sunshine Act and subsequent SUPPORT Act require public reporting by medical and technology manufacturers of payments greater than $10 in value to healthcare providers, including fees for consulting, education, research, and other reasons. For employed physicians, most institutions have rules governing consulting and speaking for industry. These may, for example, require pre-approval of consulting agreements, restrictions on speaking engagements or event attendance, or caps on payments if also serving as a principal investigator for an industry trial. Moreover, disclosure of potential conflicts of interest is essential in all settings where they may be relevant, including manuscripts, presentations or public discourse, and consenting for clinical trials.^4^^,^^5^ Professional societies are increasingly requiring disclosure of potential conflicts of committee members and may limit participation in initiatives where conflicts may pose a ­significant potential influence such as clinical guideline development.^6^

### Time commitment

As most physicians’ most limited resource is time, it is prudent to prioritize efforts with the potential for mutual benefit; for example, regular meetings with sales representatives for an established drug might be better delegated to other staff, while periodic meetings with MSLs may be of higher yield.

## How to Start and Optimize Industry Interactions

For new providers, we recommend meeting your local MSLs and sales representatives, focusing at first on those companies whose products are regularly utilized. Depending on local institutional regulations and personal preferences, these may also include food to facilitate attendance and working into a full schedule. In our practices, we or our groups’ pharmacists generally meet with commercial teams to discuss medication access when new drugs become available, and meet with MSLs periodically to review new data, pipeline developments, and discuss clinical trial involvement or investigator-initiated research proposals. Setting expectations for meeting frequency, duration, and circumstances with each representative can optimize the time commitment. Coordinating interactions with regional or national meetings can reduce clinical impact.

As the pharmaceutical toolbox for inflammatory bowel disease rapidly expands, interactions with industry are both inevitable and vital. A thoughtful approach to these interactions, mindful of potential pitfalls, will optimize the benefits of such relationships for both providers and patients.

## Data Availability

No primary data are presented in this manuscript.

## References

[1] Brax H , Fadlallah R, Al-Khaled L, et al Association between physicians’ interaction with pharmaceutical companies and their clinical practices: a systematic review and meta-analysis. PLoS One. 2017;12:e0175493. 28406971 10.1371/journal.pone.0175493 PMC5391068

[2] Fickweiler F , Fickweiler W, Urbach E. Interactions between physicians and the pharmaceutical industry generally and sales representatives specifically and their association with physicians’ attitudes and prescribing habits: a systematic review. BMJ Open. 2017;7:e016408. 10.1136/bmjopen-2017-016408 PMC5623540 28963287

[3] Mitchell AP , Trivedi NU, Gennarelli RL, et al Are financial payments from the pharmaceutical industry associated with physician prescribing?: a systematic review. Ann Intern Med. 2021;174:353-361. 33226858 10.7326/M20-5665 PMC8315858

[4] Elsolh K , Tham D, Scaffidi MA, et al Financial conflicts of interest in propensity score-matched studies evaluating biologics and biosimilars for inflammatory bowel disease. J Can Assoc Gastroenterol. 2022;5:214-220. 36196272 10.1093/jcag/gwac018 PMC9527658

[5] Khan R , Li J, Scaffidi MA, et al Conflicts of interest in inflammatory bowel disease articles on UpToDate. J Can Assoc Gastroenterol. 2021;4:10-14. 33644671 10.1093/jcag/gwz030 PMC7898378

[6] Singh S , Ananthakrishnan AN, Nguyen NH, et al AGA Clinical Guidelines Committee. Electronic Address: clinicalpractice@Gastro.org. AGA clinical practice guideline on the role of biomarkers for the management of ulcerative colitis. Gastroenterology. 2023;164:344-372. 36822736 10.1053/j.gastro.2022.12.007

